# Targeting PD-1/PD-L1 inhibits rejection in a heterotopic tracheal allograft model of lung transplantation

**DOI:** 10.3389/fphar.2023.1298085

**Published:** 2023-11-06

**Authors:** Taisuke Kaiho, Hidemi Suzuki, Atsushi Hata, Hiroki Matsumoto, Kazuhisa Tanaka, Yuichi Sakairi, Shinichiro Motohashi, Ichiro Yoshino

**Affiliations:** ^1^ Department of General Thoracic Surgery, Chiba University Graduate School of Medicine, Chiba, Japan; ^2^ Department of Medical Immunology, Chiba University Graduate School of Medicine, Chiba, Japan

**Keywords:** programmed death-1, programmed death ligand-1, lung transplantation, mouse model, immunotherapy

## Abstract

Immune checkpoint molecules such as programmed death-1 (PD-1) and programmed death ligand-1 (PD-L1) have revolutionized the field of lung cancer treatment. As part of our study, we examined the role of these proteins in acute rejection in a mouse model of heterotopic tracheal transplantation. Recipient mice were untreated (Allo group) or treated with anti-PD-L1 (aPDL1 group) or PD-L1 Fc recombinant protein (PD-L1 Fc group). A further group of C57BL/6 mice received isografts (Iso group). The occlusion rate was significantly higher in the Allo group than in the Iso group (*p* = 0.0075), and also higher in the aPD-L1 group (*p* = 0.0066) and lower in the PD-L1 Fc group (*p* = 0.030) than in the Allo group. PD-L1 Fc recombinant protein treatment significantly decreased interleukin-6 and interferon-γ levels and reduced the CD4^+^/CD8^+^ T cell ratio, without increasing PD-1 and T-cell immunoglobulin mucin 3 expression in CD4^+^ T cells. These data suggest that PD-L1 Fc recombinant protein decreases the levels of inflammatory cytokines and the proportion of CD4^+^ T cells without exhaustion. The PD-L1-mediated immune checkpoint mechanism was associated with rejection in the murine tracheal transplant model, suggesting a potential novel target for immunotherapy in lung transplantation.

## Introduction

Survival rates in patients who have undergone lung transplantation are much lower than those in patients who receive other solid organ grafts (Gracon and Wilkes, 2014). The leading cause of death more than 1 year after lung transplantation is bronchiolitis obliterans syndrome (Yusen et al., 2015). A major form of chronic lung allograft dysfunction (Glanville et al., 2019), bronchiolitis obliterans syndrome has the most significant impact on the long-term survival of lung transplant patients (Verleden et al., 2014). Treatments for chronic lung allograft dysfunction are still limited and have not substantially improved patient prognosis.

Acute rejection is a serious complication in the early phase after lung transplantation, and is also an established risk factor for the development of chronic lung allograft dysfunction (Benzimra et al., 2017).

Programmed death 1 (PD-1) was first described by Ishida et al., in 1992 (Ishida et al., 1992) and is a negative regulator that modulates T-cell activation and homeostasis. Programmed death ligand-1 (PD-L1; also known as CD274 or B7H1) is a member of the B7 family of immuno-coinhibitory and costimulatory molecules and functions as an immune checkpoint via its interaction with its receptors PD-1 and CD80.

PD-1/PD-L1 interactions play a crucial suppressive role in the immune system, attenuating autoimmunity and promoting self-tolerance by preventing T-cell activation. PD-1/PD-L1 axis targeting has been reported in other conditions, such as autoimmune diabetes (Ben Nasr et al., 2017; Ben Nas et al., 2018; Ben Nasr et al., 2022) and coronavirus disease-2019 (Loretelli et al., 2021). Furthermore, the development of immune checkpoint inhibitors, such as anti-PD-1 or anti-PD-L1 inhibitors, has revolutionized the treatment of non-small cell lung cancer (Okazaki et al., 2013; Brahmer et al., 2015; Herbst et al., 2016; Rittmeyer et al., 2017).

Associations between PD-1/PD-L1 interactions and prolonged allograft survival have been reported in murine kidney transplantation (Peng et al., 2011; Jaworska et al., 2015), heterotopic heart transplantation (Ozkaynak et al., 2002; Tanaka et al., 2007; Yang et al., 2011), liver transplantation (Morita et al., 2010), and hematopoietic cell transplantation (Al-Chaqmaqchi et al., 2013; Cassa et al., 2018). However, there are few studies on lung transplantation (Takahashi et al., 2018), and immunological tolerance has not yet been achieved through targeting of PD-1.

In this study, we investigated the role of immune checkpoint molecules in acute rejection using a murine tracheal transplant model.

## Materials and methods

### Animal model

Specific pathogen-free male inbred C57BL/6 (H-2^b^) and BALB/c (H-2^d^) mice (CLEA Japan, Inc., Tokyo, Japan) were used for heterotopic tracheal transplantation. They were housed at the Biomedical Research Center at Chiba University School of Medicine (Chiba, Japan) in accordance with institutional guidelines. BALB/c (H-2^d^) and C57BL/6 (H-2^b^) mice were used as donors and C57BL/6 (H-2^b^) mice as recipients at 8–12 weeks of age (body weight, 24–32 g).

### Surgical technique

**FIGURE 1:**
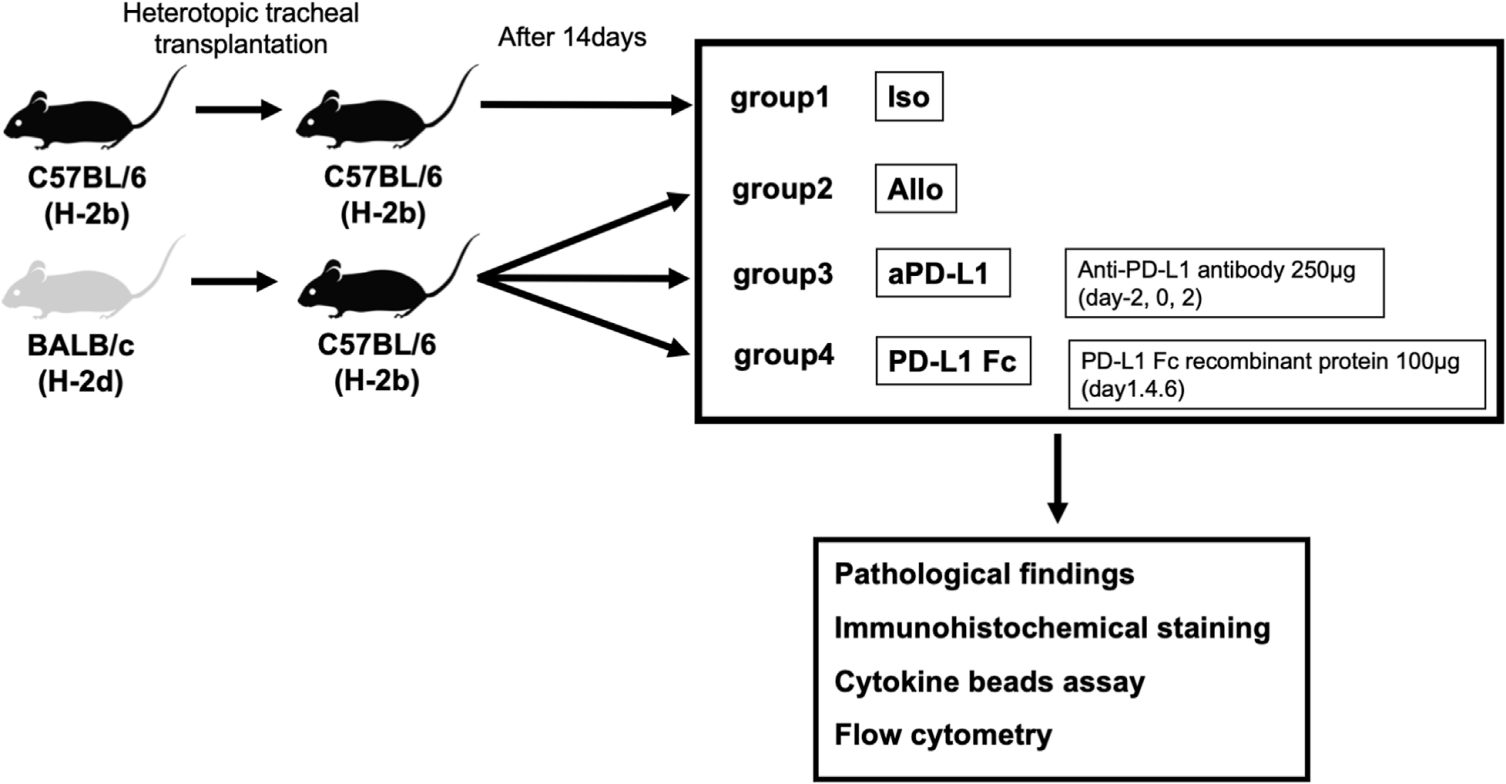
Study design. Tracheas from donor BALB/c (H-2^d^) mice were transplanted into major histocompatibility antigen-mismatched recipient C57BL/6 (H-2^b^) mice in the tracheal transplant model. Four groups (allografts (Allo), mouse anti-PD-L1 antibody (aPD-L1), PD-L1 Fc recombinant protein (PD-L1 Fc), and isografts (Iso) (n = 5)) were confirmed pathologically and analyzed. Rejection was evaluated by assessing pathologic occlusion rate, immunohistochemistry, and cytokine levels and cell surface markers by cytokine bead assay and flow cytometry, respectively. PD-L1, programmed death ligand-1.

Heterotopic tracheal transplantation was carried out as previously described (Hua et al., 2010). The transplanted tracheas were harvested on day 14 post-surgery, along with serum. We established four groups. In the Allo group, BALB/c (H-2^d^) mice were used as donors and C57BL/6 (H-2^b^) mice as recipients (n = 5). In the aPD-L1 group, recipient mice were treated intraperitoneally on days −2, 0, and 2 with 250 μg mouse anti-PD-L1 (Bio X Cell, Lebanon, NH, USA) (n = 5). In the PD-L1 Fc group, recipient mice were treated intraperitoneally on days 1, 4, and 6 with 100 μg mouse PD-L1 Fc recombinant protein (PD-L1 Fc RP; Acro Biosystems, Newark, DE, USA) (n = 5). In the Iso group, C57BL/6 (H-2^b^) mice were used as donors and as recipients (n = 5) (Figure 1).

This study was approved by the Institute for Animal Care at Chiba University (approval code: A2-015) and was performed in compliance with the Guide for the Care and Use of Laboratory Animals (National Institutes of Health publication 86–23, revised 1996). All surgical procedures were performed utilizing a sterile technique. No antibiotics were given to either donor or recipient mice. Induction of anesthesia of the donor mouse was initiated with 5% isoflurane. The mouse was then orotracheally intubated with a 20-gauge intravenous catheter and placed on a rodent ventilator, using 100% oxygen at a rate of 125 breaths/minute and approximately 0.5 mL tidal volume (2% of its body weight). The animals were maintained under general anesthesia with a mixture of isoflurane and oxygen. The donor and recipient mice were swabbed with 70% alcohol before incision. Buprenorphine (0.05–0.15 mg/kg) was administered immediately after surgery and every 8 h for 2–3 days post-surgery for analgesia, and all efforts were made to minimize suffering. Animals were monitored every day for signs of respiratory difficulty, weight loss, and the development of a moribund state. Animals were euthanized under anesthesia, and the transplanted grafts were harvested at that time. Euthanasia was performed by cervical dislocation under deep anesthesia induced by isoflurane or CO_2_.

### Histology and immunohistochemistry

Trachea grafts were harvested on day 14 post-surgery, fixed in glutaraldehyde, and paraffin embedded. Each trachea was sectioned and stained with hematoxylin/eosin and Masson’s trichrome to evaluate the presence of inflammatory cells and airway fibrosis and to diagnose acute rejection.

Luminal occlusion of the transplanted trachea was determined by measuring the area containing tissue inside the cartilage ring using ImageJ software (National Institutes of Health, Bethesda, MD, United States) (Reichenspurner et al., 1997; Schneider et al., 2012). The percentage of luminal occlusion was calculated as follows: (area within cartilage—area within residual lumen)/area within cartilage × 100%. The presence of mucus, produced by airway epithelial cells, in the lumen was not counted as occlusion. The histological changes in the respiratory epithelium were evaluated as the percentage of luminal circumference covered by ciliated epithelium (Sato et al., 2008).

Immunohistochemistry was performed with the following primary antibodies as per the manufacturer’s protocols: rabbit polyclonal anti-PD-1/CD279 antibody (catalog no. 18106-1-AP; ProteinTech Group, Rosemont, IL, USA) and polyclonal anti-PD-L1/CD274 antibody (catalog no. 17952-1-AP; ProteinTech Group).

### Serum cytokine analysis with cytometric bead array

Serum from individual representative recipient mice from each of the Iso (n = 5), Allo (n = 5), aPD-L1 (n = 5), and PD-L1 Fc (n = 5) groups was analyzed using the BD Cytometric Bead Array system (BD, Franklin Lakes, NJ, United States), as previously described (Morgan et al., 2004).

### Flow cytometry

Single cell suspensions were obtained from fresh trachea graft tissue by enzymatic digestion using collagenase type III (6000 U/mL; Worthington Biochemical Corp., Lakewood, NJ, United States) and DNase (10 mg/mL; Merck Group, Darmstadt, Germany) for 30 min at 37°C. After counting, cells were incubated with antibodies for 30 min at 4°C and analyzed using a FACSCanto II flow cytometer (BD).

Single cell suspensions were stained with fluorochrome-labeled antibodies specific for CD44 (clone IM7), CD8a (clone 53–6.7), and CD4 (clone RM45) (all from eBioscience, Inc., San Diego, CA, United States), and PD-1 (clone RMT1-30), and T-cell immunoglobulin mucin 3 (Tim-3; clone RMT3-23) (from BioLegend, San Diego, CA, United States).

### Statistical analysis

All data are presented as the mean ± standard error of the mean. When comparing two groups, data were analyzed using the Student’s t-test. All statistical analyses were performed using JMP software version 13.2 (SAS Institute, Cary, NC, United States). *p*-values <0.05 were considered statistically significant.

## Results

### Evaluation of the murine trachea transplant model

**FIGURE 2:**
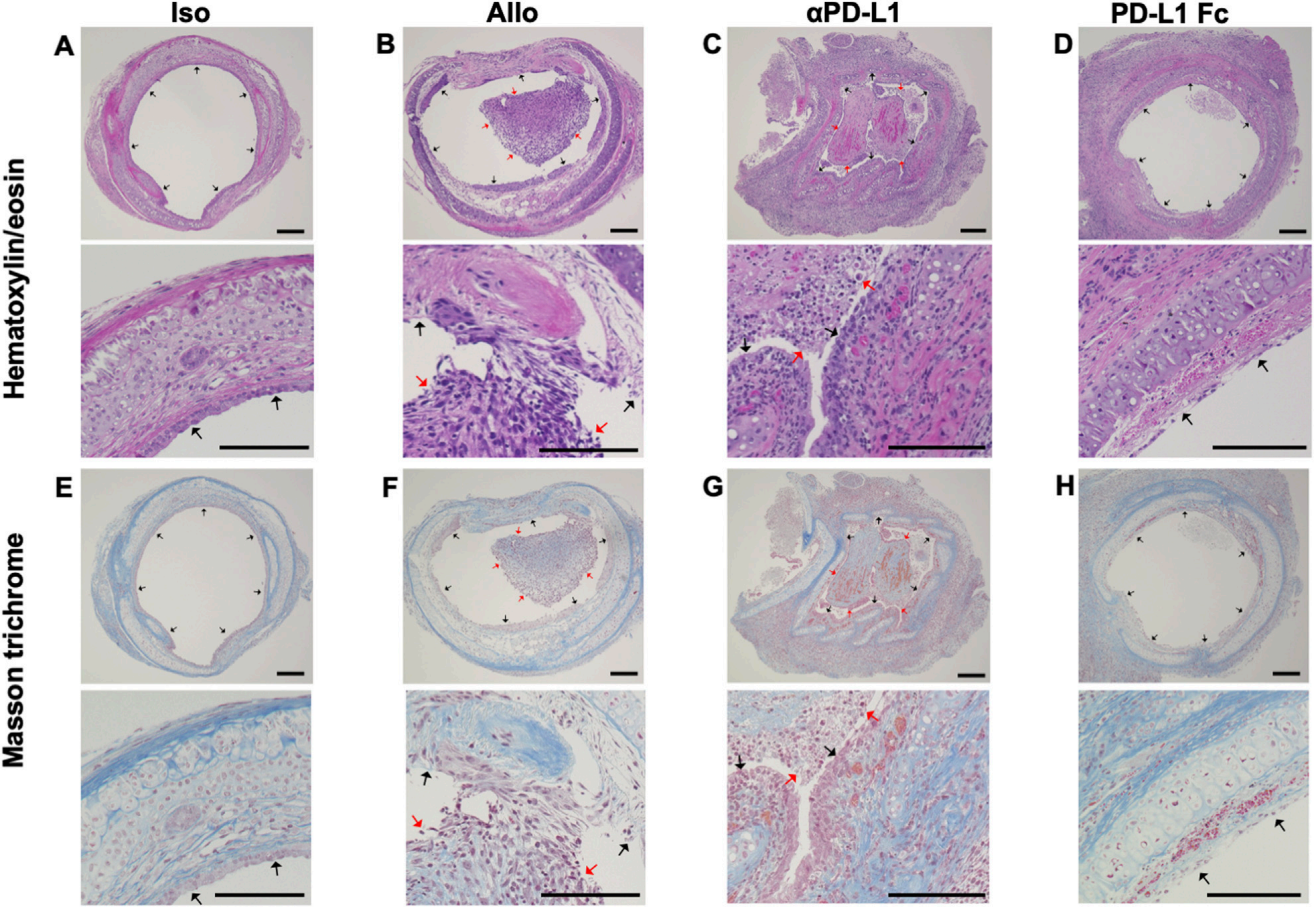
Histological features of the trachea grafts. Representative images of hematoxylin/eosin-stained tissue sections **(A–D)** (original, 10× and 40x), and Masson’s trichrome-stained tissue sections **(E–H)** in the Allo **(A and E)**, aPD-L1 **(B and F)**, PD-L1 Fc **(C and G)**, and Iso **(D and H)** groups (original, 10× and 40x). aPD-L1, anti-programmed death ligand-1; PD-L1, programmed death ligand-1. Black arrows indicate epithelium and red arrows indicate locations of cellular infiltration. Scale bar = 500 μm.

Trachea graft sections were stained with hematoxylin/eosin (Figures 2A–D) or Masson’s trichrome (Figures 2E–H). The airway was not obstructed by fibrotic tissue in the Iso group (Figures 2A,E). The Allo group showed airway occlusion with luminal cell infiltration and fibrotic tissue, which was deposition of mononuclear cells in the graft/denuded epithelium as evidence of acute rejection (Figures 2B,F). The airway occlusion was greater in the aPD-L1 group (Figures 2C,G) and less in the PD-L1 Fc group (Figures 2D,H) than in the Allo group.

**FIGURE 3:**
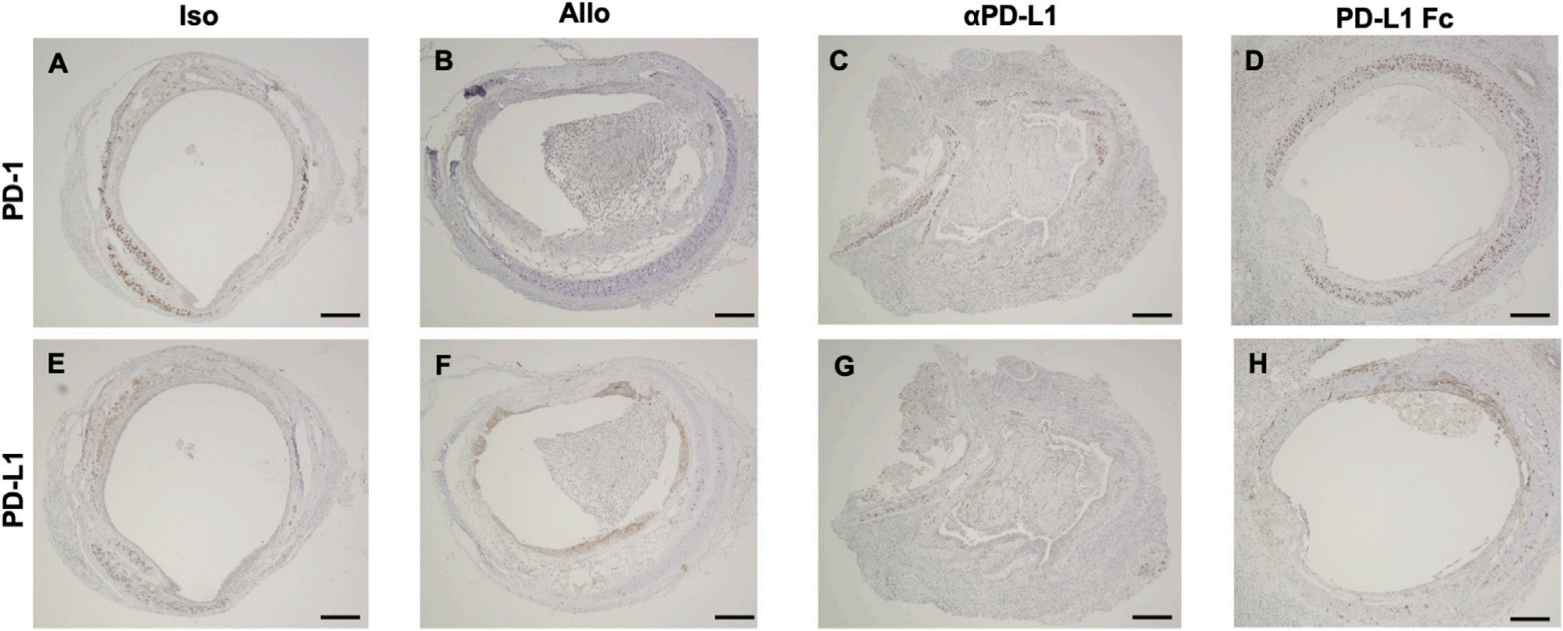
Immunohistochemical features of trachea grafts. Representative images of PD-1 immunohistochemical staining **(A–D)** and PD-L1 immunohistochemical staining **(E–H)** in the Allo **(A and E)**, aPD-L1 **(B and F)**, PD-L1 Fc **(C and G)**, and Iso **(D and H)** groups. aPD-L1, anti-programmed death ligand-1; PD-1, programmed death-1; PD-L1, programmed death ligand-1. Scale bar = 500 μm.

Trachea graft sections were also stained using antibodies against PD-1 (Figures 3A–D) and PD-L1 (Figures 3E–H). Immunohistochemistry revealed that luminal infiltrating cells were positive for PD-1 (Figures 3B,C,I,J) and PD-L1 (Figures 3F,G) in the Allo and aPD-L1 groups.

**FIGURE 4:**
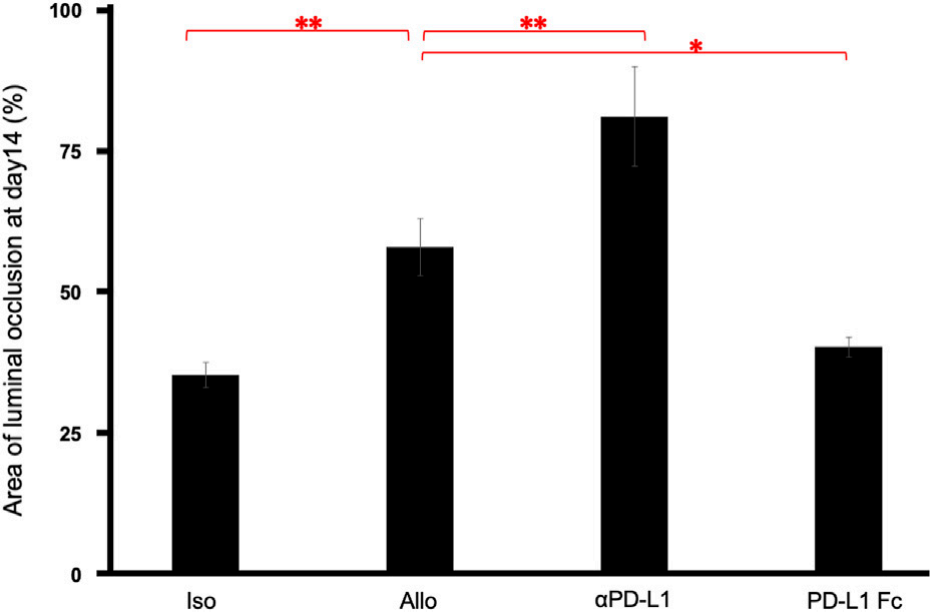
Pathological occlusion rate. The occlusion rate was significantly higher in the Allo group than in the Iso group (58.24% ± 5.02% vs. 35.58% ± 2.17%, *p* = 0.0075), higher in the aPD-L1 group than in the Allo group (81.36% ± 8.77% vs. 58.24% ± 5.02%, *p* = 0.0066), and lower in the PD-L1 Fc group than in the Allo group (40.54% ± 1.76% vs. 58.24% ± 5.02%, *p* = 0.0297) (n = 5). *p < 0.05, **p < 0.01. aPD-L1, anti-programmed death ligand-1; PD-L1, programmed death ligand-1.

The luminal occlusion rate was significantly higher in the Allo group than in the Iso group (58.24% ± 5.02% vs. 35.58% ± 2.17%, *p* = 0.0075), higher in the aPD-L1 group than in the Allo group (81.36% ± 8.77% vs. 58.24% ± 5.02%, *p* = 0.0066), and lower in the PD-L1 Fc group than in the Allo group (40.54% ± 1.76% vs. 58.24% ± 5.02%, *p* = 0.030) (Figure 4).

### PD-L1 Fc RP suppressed production of inflammatory cytokines

**FIGURE 5:**
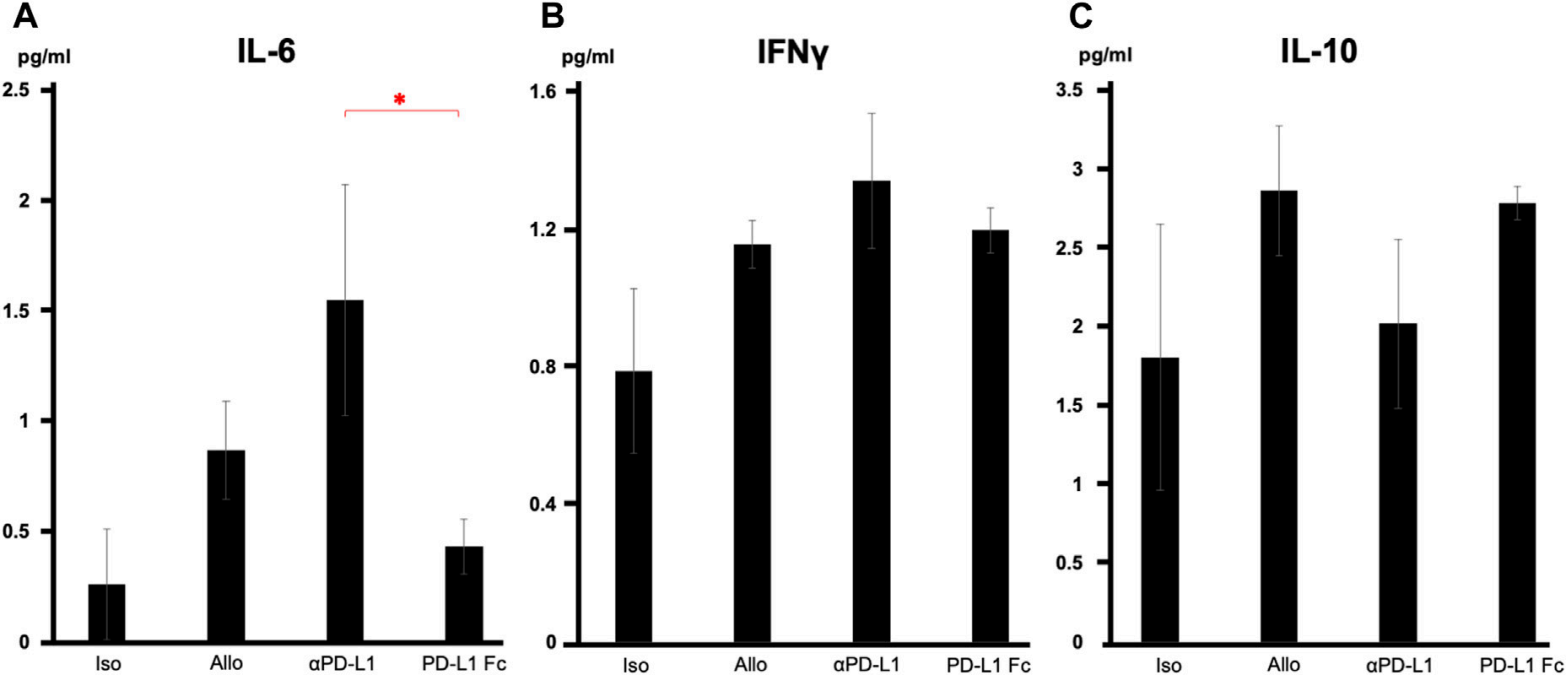
Serum cytokine levels. **(A)** IL-6 levels were significantly suppressed in the PD-L1 Fc group but increased in the aPD-L1 group (0.43 ± 0.12 pg/mL vs. 1.55 ± 0.52 pg/mL, *p* = 0.0366). **(B)** A similar trend was observed in IFN-γ in the four groups. **(C)** IL-10 levels were increased in the Allo (2.87 ± 0.41 pg/mL) and PD-L1 Fc (2.79 ± 0.10 pg/mL) groups and suppressed in the aPD-L1 group (2.02 ± 0.53 pg/mL) (n = 5). *p < 0.05. aPD-L1, anti-programmed death ligand-1; IFN-γ, interferon-γ; IL, interleukin; PD-L1, programmed death ligand-1.

Serum cytokine analysis revealed that interleukin-6 levels were significantly suppressed in the PD-L1 Fc group, whereas they increased in the aPD-L1 group (0.43 ± 0.12 pg/mL vs. 1.55 ± 0.52 pg/mL, *p* = 0.037) (Figure 5A). Interferon-γ levels were also suppressed in the PD-L1 Fc group (1.20 ± 0.06 pg/mL) and increased in the aPD-L1 group (1.34 ± 0.20 pg/mL), although not significantly compared with the Allo group (Figure 5B). In contrast, levels of interleukin-10, a cytokine with potent anti-inflammatory properties, were increased in the Allo group (2.87 ± 0.41 pg/mL) and the PD-L1 Fc group (2.79 ± 0.10 pg/mL) and suppressed in the aPD-L1 group (2.02 ± 0.53 pg/mL) (Figure 5C).

### PD-L1 Fc RP inhibits CD4^+^ T-cell proliferation

**FIGURE 6:**
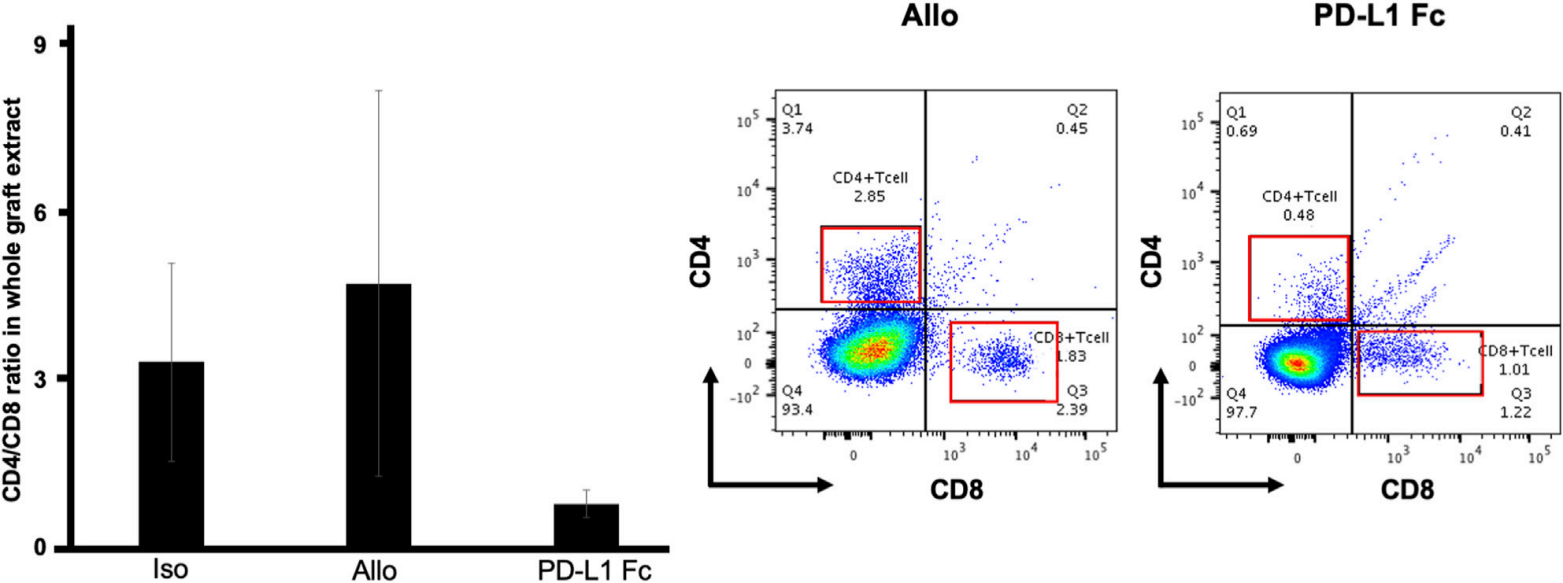
CD4^+^/CD8^+^ T cell ratio. In flow cytometric analyses of whole tracheal tissue, the CD4^+^/CD8^+^ ratio was lower in the PD-L1 Fc group (0.78 ± 0.25) than in the Allo group (4.74 ± 3.47) and the Iso group (3.33 ± 1.78) (n = 3). This ratio was increased in the Allo group compared with the Iso group. PD-L1, programmed death ligand-1.

In flow cytometric analyses of whole tracheal tissue, the CD4^+^/CD8^+^ ratio was lower in the PD-L1 Fc group (0.78 ± 0.25) than in the Allo group (4.74 ± 3.47) and the Iso group (3.33 ± 1.78) (Figure 6) (n = 3).

### PD-L1 Fc RP did not increase the expression of PD-1 and Tim-3 in CD4^+^CD44^+^ T cells

CD44 is an activation marker that distinguishes memory and effector T cells from their naïve counterparts (Schumann et al., 2015). CD4^+^CD44^+^ T cells are antigen-stimulated memory T cells. Both PD-1 and Tim-3 are exhaustion markers and can function as negative regulators of T-cell responses. CD4^+^CD44^+^ T cells were analyzed for expression of the immune checkpoint molecules PD-1 and Tim-3. In this analysis, cells that coexpressed PD-1 and Tim-3 represented the most exhausted fraction.

**FIGURE 7:**
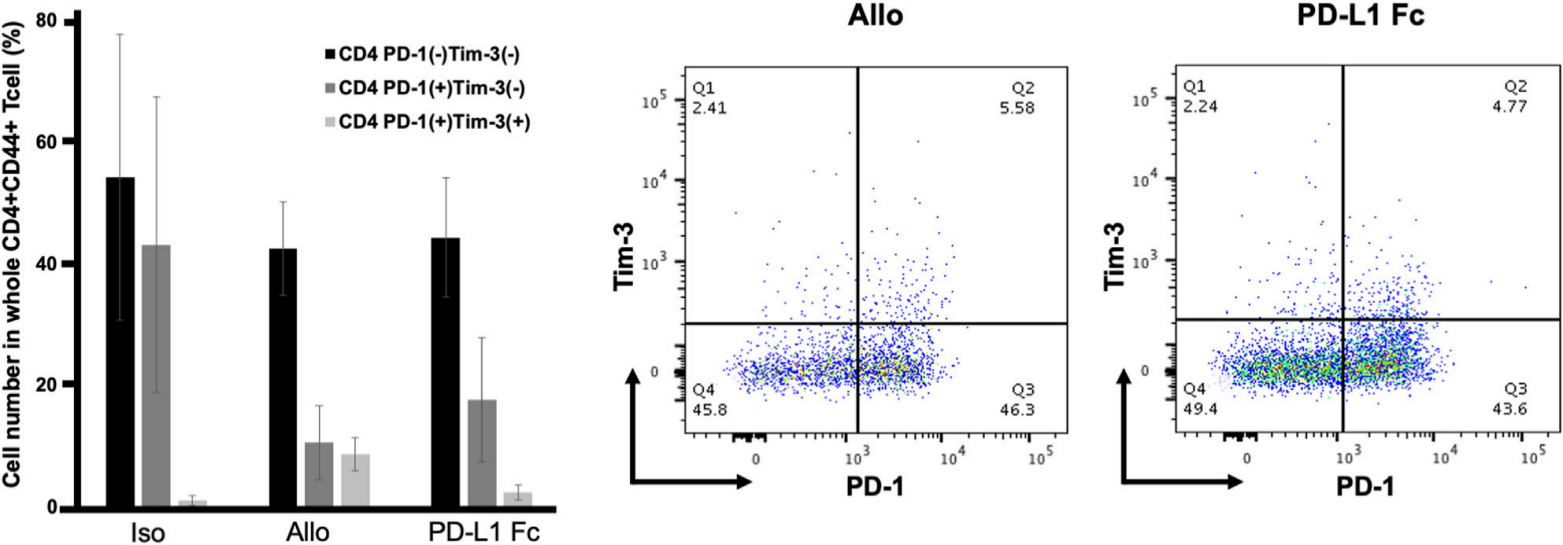
Proportions of CD4^+^CD44^+^ T cells expressing PD-1 and Tim3. The proportion of CD4^+^CD44^+^ T cells that were PD-1^-^/Tim-3^-^, PD-1^+^/Tim-3^-^, and PD-1^+^/Tim3^+^ was not significantly different in the PD-L1 Fc group (44.3% ± 9.8%, 17.6% ± 10.2%, and 2.4% ± 1.2%, respectively) compared to the Allo group (42.5% ± 7.7%, 10.6% ± 6.1%, and 8.7% ± 2.7%, respectively) (n = 3). PD-1, programmed death-1; PD-L1, programmed death ligand-1; Tim-3, T-cell immunoglobulin mucin 3.

The proportion of CD4^+^CD44^+^ T cells that were PD-1^-^/Tim-3^-^, PD-1^+^/Tim-3^-^, and PD-1^+^/Tim3^+^ was not significantly different in the PD-L1 Fc group (44.3% ± 9.8%, 17.6% ± 10.2%, and 2.4% ± 1.2%, respectively) compared to the Allo group (42.5% ± 7.7%, 10.6% ± 6.1%, and 8.7% ± 2.7%, respectively) (Figure 7).

## Discussion

This study examined the role of PD-1/PD-L1 in acute rejection using a murine tracheal transplant model. The administration of PD-L1 Fc RP decreased inflammatory cytokine levels and inhibited CD4^+^ T-cell proliferation; allograft airway fibrosis was also suppressed. In cancer immunotherapy, PD-1/PD-L1 blockade activates the PD-1/PD-L1 axis, which serves as a mechanism for tumor evasion of host tumor antigen-specific T-cell immunity. This study was initiated to investigate the role of immune checkpoint molecules in lung transplantation and their potential therapeutic applications.

The main finding of the present study was that PD-1/PD-L1 was associated with transplant rejection in the tracheal transplant model. In our study, administration of PD-L1 Fc RP suppressed airway occlusion by luminal cell infiltration and fibrotic tissue, suggesting that acute rejection could be regulated by targeting this axis. These results were consistent with those described in other transplant models.

The present study is, to the best of our knowledge, the first to report the potential utility of PD-1/PD-L1 for the treatment of lung transplant rejection in a murine heterotopic tracheal transplant model. Takahashi et al. reported that PD-1 expression regulates the differentiation of CD8^+^ T cells within lung allografts (Takahashi et al., 2018), but the effect of PD-1 targeting therapy was not mentioned. The results of this study provide a first step toward the treatment of lung transplant rejection by targeting immune checkpoint molecules. T-cell immunoglobulin and ITIM domain is another immune checkpoint molecule, and it has been reported that administering T-cell immunoglobulin and ITIM domain-Fc recombinant protein suppressed the proliferation of T cells and interferon-γ production in a mouse model (Yu et al., 2009). Other immune checkpoint molecules, such as lymphocyte activation gene-3 or Tim-3, also modulate the immune system. Combined blockade of the Tim-3 and PD-1 pathways has been reported to be a promising strategy for tumor immunotherapy (Tian and Li, 2021). These immune checkpoint molecules may possibly be of use in transplant rejection therapy (McGrath and Najafian, 2012).

Another important finding of the present study is that PD-1/PD-L1 was associated with suppression of the inflammatory cytokines, interleukin-6 and interferon-γ, and a decrease in the proportion of CD4^+^ T cells without exhaustion. Treatment with PD-L1 Fc RP did not increase the expression of Tim-3 in CD4^+^CD44^+^ T cells. This result may provide insight into the mechanism of allograft rejection in lung transplantation and provide new options for the targeting of immune checkpoint inhibitors in tumor immunology. Further *in vivo* studies are required to establish the molecular mechanisms of immune checkpoints in therapeutic approaches, including their association with CD4+Foxp3+ T cells. Translational research on immune checkpoint molecules in lung cancer is currently being performed (Kamada et al., 2019), and there is potential for clinical applications of immune checkpoint molecules for lung allograft rejection in the future.

The current study has some limitations. First, this murine heterotopic tracheal transplant model is an acute rejection model with a major histocompatibility mismatch. Heterotopic tracheal transplant model is a high-throughput procedure useful for studying the cellular requirements for acute rejection models. Histological changes also make this model especially valuable for studying chronic rejection observed in the airways of human lungs (Lama et al., 2017). The tracheal transplant model has disadvantages including no ventilation of the transplanted trachea, inhibition of mucociliary clearance and retained secretions, and differences in the microenvironment. To analyze acute rejection more precisely, a murine orthotopic lung transplant model should be utilized. This model was developed by Okazaki et al., 2007, and a murine obliterative bronchiolitis orthotopic lung transplant model has also been established (Suzuki et al., 2012; Hata et al., 2020). Orthotopic lung transplant models are a closer reflection of the clinical situation than heterotopic lung transplant models and will therefore be essential to understand the mechanisms underlying the pathogenesis of rejection in lung transplantation.

Second, cellular rejection was mainly analyzed in this study of acute rejection; however, humoral rejection may also be involved. The association between antibody-mediated rejection and C4d staining analysis (Levine et al., 2016) was not investigated in this study and will be further explored in the future.

Finally, the PD-1/PD-L1 mechanism involved in acute rejection following lung transplantation remains unidentified. In the present study, PD-L1 Fc RP was found to upregulate PD-L1 levels; however, it has been reported that overexpression of PD-L1 in dendritic cells inhibits allogeneic lymphocyte activation in mice (Peng et al., 2011; Li et al., 2012). This mechanism suggests the possibility of donor-specific immune tolerance, which has been reported in other murine organ transplant models but has not been reported in the lung transplant model. This donor-specific treatment might be a potential therapeutic modality for rejection in lung transplantation.

In conclusion, the PD-L1-mediated immune checkpoint mechanism was associated with rejection, which suggests a potential novel target for immunotherapy in lung transplantation. PD-1/PD-L1 should be further explored as a potential therapeutic modality for rejection.

## Data Availability

The raw data supporting the conclusion of this article will be made available by the authors, without undue reservation.

## Abbreviations

PD-1, programmed death-1; PD-L1, programmed death ligand-1; RP, recombinant protein; Tim-3, T-cell immunoglobulin mucin 3.
